# Adenosine Receptors Modulate the Exogenous Ketogenic Supplement-Evoked Alleviating Effect on Lipopolysaccharide-Generated Increase in Absence Epileptic Activity in WAG/Rij Rats

**DOI:** 10.3390/nu13114082

**Published:** 2021-11-15

**Authors:** Brigitta Brunner, Csilla Ari, Dominic P. D’Agostino, Zsolt Kovács

**Affiliations:** 1 Faculty of Sciences, Institute of Biology, University of Pécs, Ifjúság Str. 6, 7624 Pécs, Hungary; brunnerb28@gmail.com; 2 Savaria University Centre, Department of Biology, ELTE Eötvös Loránd University, Károlyi Gáspár tér 4, 9700 Szombathely, Hungary; zskovacsneuro@gmail.com; 3 Ketone Technologies LLC, Tampa, FL 33612, USA; ddagosti@usf.edu; 4 Behavioral Neuroscience Research Laboratory, Department of Psychology, University of South Florida, Tampa, FL 33620, USA; 5 Laboratory of Metabolic Medicine, Department of Molecular Pharmacology and Physiology, Morsani College of Medicine, University of South Florida, Tampa, FL 33612, USA; 6 Institute for Human and Machine Cognition, Ocala, FL 34471, USA

**Keywords:** ketogenic supplements, ketosis, adenosine receptors, LPS, absence epilepsy, WAG/Rij rats

## Abstract

It has been previously demonstrated that KEKS food containing exogenous ketogenic supplement ketone salt (KS) and ketone ester (KE) decreased the lipopolysaccharide (LPS)-generated increase in SWD (spike-wave discharge) number in Wistar Albino Glaxo/Rijswijk (WAG/Rij) rats, likely through ketosis. KEKS-supplemented food-generated ketosis may increase adenosine levels, and may thus modulate both neuroinflammatory processes and epileptic activity through adenosine receptors (such as A1Rs and A2ARs). To determine whether these adenosine receptors are able to modify the KEKS food-generated alleviating effect on LPS-evoked increases in SWD number, an antagonist of A1R DPCPX (1,3-dipropyl-8-cyclopentylxanthine; 0.2 mg/kg) with LPS (50 µg/kg) and an antagonist of A2AR SCH58261 (7-(2-phenylethyl)-5-amino-2-(2-furyl)-pyrazolo-[4,3-e]-1,2,4-triazolo[1,5-c]pyrimidine; 0.5 mg/kg) with LPS were co-injected intraperitoneally (i.p.) on the ninth day of KEKS food administration, and their influence not only on the SWD number, but also on blood glucose, R-beta-hydroxybutyrate (R-βHB) levels, and body weight were measured. We showed that inhibition of A1Rs abolished the alleviating effect of KEKS food on LPS-generated increases in the SWD number, whereas blocking A2ARs did not significantly modify the KEKS food-generated beneficial effect. Our results suggest that the neuromodulatory benefits of KEKS-supplemented food on absence epileptic activity are mediated primarily through A1R, not A2AR.

## 1. Introduction

Digestion of administered exogenous ketogenic supplements (EKSs), such as ketone salts and ketone esters (KSs and KEs, respectively) can liberate ketone bodies, such as beta-hydroxybutyrate (βHB) and acetoacetate (AcAc) [1]. These molecules can transport to the bloodstream and are able to evoke an increase in ketone body levels (therapeutic ketosis) without dietary restriction, such as a ketogenic diet [2,3]. After that, ketone bodies may be transported to different cells (such as neurons and glia) through monocarboxylate transporters and can be readily used as an energy source in the mitochondria [4,5]. Indeed, adaptation of the brain metabolism to ketone bodies as a main source of fuel was demonstrated [6,7]. It is widely accepted that ketone bodies (e.g., βHB), are not only a potential energy source for different tissues of the body, but also signaling molecules for the central nervous system’s processes [4,5]. Moreover, administration of EKSs can significantly increase and sustain the blood level of ketone bodies, which is a safe and well-tolerated method to evoke nutritional ketosis [2,3]. Thus, EKSs may be able to alleviate symptoms of different central nervous system diseases by elevating ketones independent of changing dietary macronutrient composition [2,3,8]. Indeed, EKSs showed therapeutic potential, for example, in the treatment of anxiety [2,9], different types of seizure disorders [10,11], amyotrophic lateral sclerosis [12,13], as well as Alzheimer’s disease and Parkinson’s disease [14,15]. However, despite the potential beneficial effects of EKS administration in the treatment of central nervous system disorders, their mechanism(s) of action are largely unknown.

It was suggested that EKS-evoked ketosis can attenuate inflammatory processes [16]. Moreover, inflammation may have a role in the pathomechanism of absence epilepsy [17,18]. Consequently, EKSs may be able to generate alleviating influences on absence epileptic activity through modulation of inflammatory processes. Indeed, administration of EKSs decreased not only spontaneous absence epileptic seizures [11], but also lipopolysaccharide (LPS)-generated increases in the number of spike-wave discharges (SWDs) [19] in a rat model of human absence epilepsy (Wistar Albino Glaxo/Rijswijk rat: WAG/Rij rat). In this model, the focal onset of seizures in the cortex (cortical focus) was triggered and the genesis caused rapid propagation in SWDs in electroencephalograms (EEG) [20,21]. It was also demonstrated that inflammatory processes can precipitate and aggravate epileptic seizures [22], for example in WAG/Rij rats [17,18,23] likely via Toll-like receptor 4 (TLR4)/interleukin-1β (IL-1β)/cyclooxygenase-2 (COX-2)/prostaglandin E_2_ (PGE_2_) system [22,23,24,25], leading to increased cortical excitability and thereby enhancing absence epileptic activity in WAG/Rij rats [17,23]. Moreover, it has also been suggested that adenosine has a role in the modulation of neuroinflammatory processes via its receptors, such as inhibitory (A1) and excitatory (A2A) adenosine receptors (A1Rs and A2ARs, respectively) [26,27], which receptors (and the adenosinergic system) are implicated in the pathomechanism of absence epilepsy [28,29], at least in WAG/Rij rats. Since EKSs-evoked ketosis may increase the adenosine level [30], we hypothesized that A1Rs and/or A2ARs may modulate the EKSs-evoked beneficial influences on LPS-generated aggravation in absence epileptic seizures (SWD number). Thus, we investigated the effect of an A1R antagonist (DPCPX: 1,3-dipropyl-8-cyclopentylxanthine) and an A2AR antagonist (SCH58261: 7-(2-phenylethyl)-5-amino-2-(2-furyl)-pyrazolo-[4,3-e]-1,2,4-triazolo[1,5-c]pyrimidine) on generated alleviating influence of ketone-supplemented food (KEKS food) on LPS-evoked increase in SWD number in WAG/Rij rats.

## 2. Materials and Methods

### 2.1. Animals

Animal treatments and surgeries were carried out based on the guidelines of the Hungarian Act of Animal Care and Experimentation (1998, XXVIII, Section 243), European Communities Council Directive 24 November 1986 (86/609/EEC) and EU Directive 2010/63/EU (license number VA/ÉBNTF02/85-8/2016; National Scientific Ethical Committee on Animal Experimentation, Hungary). Pain and suffering, as well as the number of experimental animals were minimized.

WAG/Rij male rats (10 months old, 304–328 g; breeding colony of rats at ELTE Savaria University Centre, Szombathely, Hungary) were used. Animals were housed in groups (containing 3–4 rats per group) under standard laboratory conditions (we provided a 12:12 h light–dark cycle; light was on: 08.00 a.m.–08.00 p.m.; free access to water and food; air-conditioned rooms: 22 ± 2 °C).

### 2.2. Electrode Implantation

Electrode implantation for EEG recording was carried out under isoflurane anesthesia (Isoflurane was 2.0–2.5% in an Isoflurane–air mixture) [31]. Stainless steel screw electrodes were implanted epidurally above the frontal cortex and parietal cortex (based on stereotaxic coordinates: AP 2.0 mm, L 2.1 mm and AP −6.5 mm, L 2.1 mm, respectively) [32]. Ground screw electrodes and reference electrodes were implanted above the cerebellar cortex [33]. Electrodes were soldered to a ten-pin socket. Finally, dentacrylate cement (Ivoclar, Liechtenstein) was used to fix the electrodes to the skull. To alleviate post-operative pain, lidocaine ointment (5%; EGIS, Hungary) was used.

### 2.3. EEG Recording and Evaluation

Rats (*n* = 33) were allowed to recover from surgery for 2 weeks (recovery period) before the recording of EEG. EEG recording was carried out by the Bioamp4 differential amplifier (Supertech Ltd., Pécs, Hungary) and the CED 1401 mkII device (Cambridge Electronic Design Ltd., UK, Cambridge) between 1.30 p.m. and 4.00 p.m. (sampling rate: 500 Hz; bandwidth of the EEG recording: 0.3 Hz to 150 Hz) [34].

As handling may evoke stress, which can induce changes in both the behavior of rats and, thereby, the SWD number for about 20–25 min [20,23,34], we evaluated the SWD number between 30 and 150 min of recording periods similar to previous studies [11,19]. However, it was also observed [23,35] that behavioral changes and its effect on SWD number disappeared within 25–30 min after treatments. One-hour sections of EEG recordings were evaluated separately [23]. SWDs were separated manually from the EEG based on their main features (SWDs consist a train of asymmetric spikes and slow waves; discharge frequency within SWDs: 7–11 Hz; averaged SWD duration: 1–30 s) [20].

### 2.4. Administration of Exogenous Ketogenic Supplements and Different Drugs

It has been previously demonstrated that *ad libitum* feeding of rats by ketone-supplemented food with a paste-like consistency (KEKS food: 10% KE/R,S-1,3-butanediol—acetoacetate diester and 10% KS/Na^+^ and Ca^2+^-ketone salt, % by weight, were mixed with powdered standard rodent chow, 1% saccharine and water) increased blood ketone body level and decreased the number of spontaneously developed SWDs between 6–9 days of administration without side effects in WAG/Rij rats [19]. It was also demonstrated that KEKS food also decreased the LPS-generated increase in SWD number on the ninth day of KEKS food administration in WAG/Rij rats [19]. Consequently, we fed the animals with KEKS food for nine days. Moreover, based on our previous results [11,23,31,34] on WAG/Rij rats, we intraperitoneally (i.p.) administered the effective and well-tolerated dose of LPS (50 µg/kg; Sigma-Aldrich, Inc., Hungary) alone; LPS with both a non-proepileptic dose of an A1R antagonist DPCPX (0.2 mg/kg; Sigma-Aldrich, Inc., Hungary) (DPCPX + LPS) and non-antiepileptic dose of an A2AR antagonist SCH58261 (0.5 mg/kg; Sigma-Aldrich, Inc., Hungary) (SCH58261 + LPS); and KEKS food with LPS (KEKS food + LPS), with DPCPX and LPS (KEKS food + DPCPX + LPS) and SCH58261 and LPS (KEKS food + SCH58261 + LPS). As 1–30% dimethyl sulfoxide (DMSO) solution did not change absence epileptic activity in WAG/Rij rats [36], both DPCPX and SCH58261 were dissolved in 10% DMSO (Sigma-Aldrich Inc., Hungary) solution, whereas LPS was administered in saline [23,31].

### 2.5. Treatments and Animal Groups

**Figure 1:**
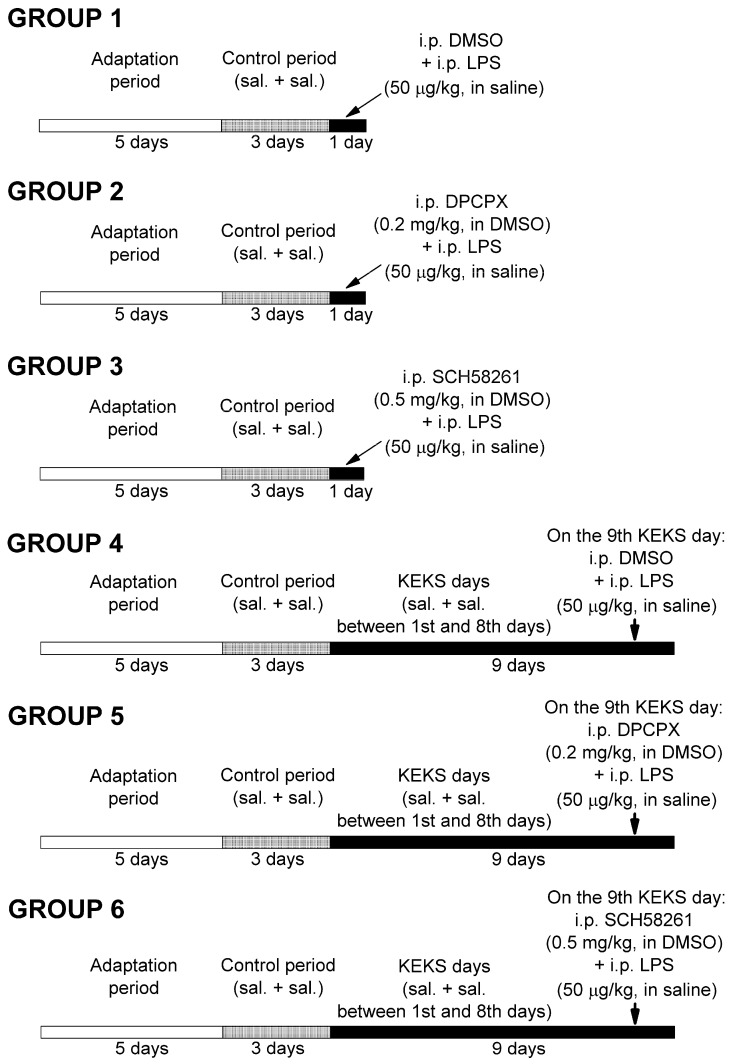
Experimental design. Abbreviations: DMSO, dimethyl sulfoxide; DPCPX, 1,3-dipropyl-8-cyclopentylxanthine; i.p., intraperitoneal; KEKS days, days KEKS food (10% ketone ester/KE, 10% ketone salt/KS and 1% saccharin in paste-like standard rodent chow) administration; LPS, lipopolysaccharide; sal. + sal.: i.p. 0.3 mL saline/100 g body weight (first injection) + 0.3 mL saline/100 g body weight (second injection; 30 min later); SCH582361, 7-(2-phenylethyl)-5-amino-2-(2-furyl)-pyrazolo-[4,3-e]-1,2,4-triazolo[1,5-c]pyrimidine.

After the recovery period, rats were assigned into six groups (Figure 1). For the adaptation of animals to the experimental procedures, rats were handled and EEG recordings were carried out every day for 5 days. During these 5 days (adaptation period), animals were fed by powdered standard rodent chow, which was mixed with water and 1% saccharine (paste-like standard rodent chow without KEKS) for adaptation of animals not only to EEG recordings, but also to paste-like food. Then, in order to determine the averaged control SWD number, all of the rats were further fed with paste-like standard rodent chow (without KEKS) on three consecutive days. Moreover, all rats were i.p. injected by saline (first injection; 0.3 mL/100 g body weight) and it was followed by the same saline injection (30 min later; second injection; 0.3 mL/100 g body weight) on the three-day control period, and the EEG recording was carried out (three-day control period).

After three-day control periods, animals in groups 1–3 received two i.p. injections (first injections were followed by second injections 30 min later) on the fourth day of the experiments, and EEGs were recorded (Figure 1). In relation to group 1 (*n* = 5), 10% DMSO solution (0.3 mL/100 g body weight; first injection) and LPS (50 µg/kg) in 0.3 mL saline/100 g body weight (second injection) were injected. A combined injection of DPCPX (first injection; i.p. 0.2 mg/kg in 0.3 mL 10% DMSO solution/100 g body weight) with LPS (second injection; 50 µg/kg in 0.3 mL saline/100 g body weight *n* = 5), as well as SCH58261 (first injection; i.p. 0.5 mg/kg in 0.3 mL 10% DMSO solution/100 g body weight) with LPS (second injection; 50 µg/kg in 0.3 mL saline/100 g body weight *n* = 5) were used in groups 2 and 3, respectively.

After control periods, rats in groups 4, 5, and 6 were fed with KEKS food for 9 consecutive days (KEKS days) (Figure 1) and were i.p. injected by 0.3 mL saline/100 g body weight (first injection) and by same injections (second injection; 0.3 mL saline/100 g body weight, 30 min later) between the first and eighth days followed by EEG recording. It was previously demonstrated that KEKS food decreased both the number of spontaneously developed SWDs and LPS-generated increase in SWD number between 6–9 days of treatment [19]. However, to strengthen the beneficial effect of KEKS food on LPS-induced enhancement of the SWD number [19], animals in group 4 (*n* = 6) were injected with 0.3 mL 10% DMSO solution/100 g body weight (first injection) and, 30 min later, with LPS (second injection; 50 µg/kg in 0.3 mL saline/100 g body weight) on the ninth day of KEKS food treatment. Moreover, to investigate the putative effects of combined administration of DPCPX with LPS and SCH58261 with LPS on the SWD number in KEKS-treated animals, in a recent study, rats (group 5 and group 6) received two i.p. injections on the ninth day of KEKS food treatment (these injections were similar to injections used in group 2 and group 3 on the fourth day of the experiments). Namely, in group 5 (*n* = 6), first i.p. injection (0.3 mL 10% DMSO solution/100 g body weight) contained DPCPX (0.2 mg/kg), whereas the second injection contained LPS (50 µg/kg in 0.3 mL saline/100 g body weight). In group 6 (*n* = 6), the first i.p. injection was 0.5 mg/kg SCH58261 (in 0.3 mL 10% DMSO solution/100 g body weight), whereas the second i.p. injection contained 50 µg/kg LPS (in 0.3 mL saline/100 g body weight). EEGs were recorded every day. As it has been demonstrated previously that KEKS food alone decreased the SWD number [19], whereas i.p. 0.2 mg/kg DPCPX and i.p. 0.5 mg/kg SCH58261 alone did not change the SWD number in WAG/Rij rats [11,31], these experiments were not carried out again in this study.

### 2.6. Measuring the Level of Blood Glucose and R-βHB, and Body Weight

Blood was taken from the tail vein of rats. We used a glucose and ketone monitoring system (Precision Xtra™, Abbott Laboratories, Irving, TX, USA) to measure the blood glucose (mg/dL) and βHB (R-βHB; mmol/L) levels [2,11]. This equipment only detects blood levels of R-βHB. Consequently, the total blood ketone levels (R-βHB + L-βHB + AcAc + acetone) would be higher than was detected in this study. Blood glucose and R-βHB levels were measured on the third control day (control) and on the first and the ninth days of KEKS food treatment (groups 4–6). We also measured the body weight of rats before KEKS food treatment began (third control day, control) and after the last (ninth) KEKS administration day (groups 4–6).

### 2.7. Statistical Analysis

It was previously demonstrated that administration of EKSs (such as KE and KEKS food), i.p. LPS (50 µg/kg), DPCPX (0.2 mg/kg) and SCH58261 (0.5 mg/kg) had no influence on sleep-waking stages, average SWD time, and discharge frequency within SWDs in WAG/Rij rats [11,19,23,28,31,34]. Thus, in a recent study we focused on SWD number alteration generated by LPS (i.p. 50 µg/kg) alone (group 1), by combined administration of DPCPX (i.p. 0.2 mg/kg) with LPS (i.p. 50 µg/kg) (group 2), SCH58261 (i.p. 0.5 mg/kg) with LPS (i.p. 50 µg/kg) (group 3) and KEKS food with LPS (i.p. 50 µg/kg) (group 4). However, until now we had no data on the presumptive effects of combined administration of KEKS food with DPCPX and LPS, as well as KEKS food with SCH58261 and LPS (groups 5 and 6, respectively) not only on the SWD number, but also on SWD time. Thus, in relation to groups 5 and 6, the average SWD time and total SWD time were also investigated on the ninth KEKS treatment day between 30–90 min.

All results were expressed as means ± SEM (standard error of the mean). The pre-treatment control SWD numbers (groups 1–6) and average SWD time (groups 5 and 6) were the grand average counted from the results of control days (three-day control period). In relation to the blood R-βHB and glucose level, as well as body weight, the results were counted from the values determined on the last (third) control days. For data analysis, GraphPad PRISM 9 software was used. Significance was determined by one- or two-way analysis of variance (ANOVA), Tukey’s multiple comparisons test, and Šídák’s multiple comparisons test [2]. Statistical significance was considered at *p* < 0.05.

## 3. Results

### 3.1. Effect of Combined Administration of DPCPX with LPS and SCH58261 with LPS on LPS-Evoked Increase in SWD Number

**Figure 2:**
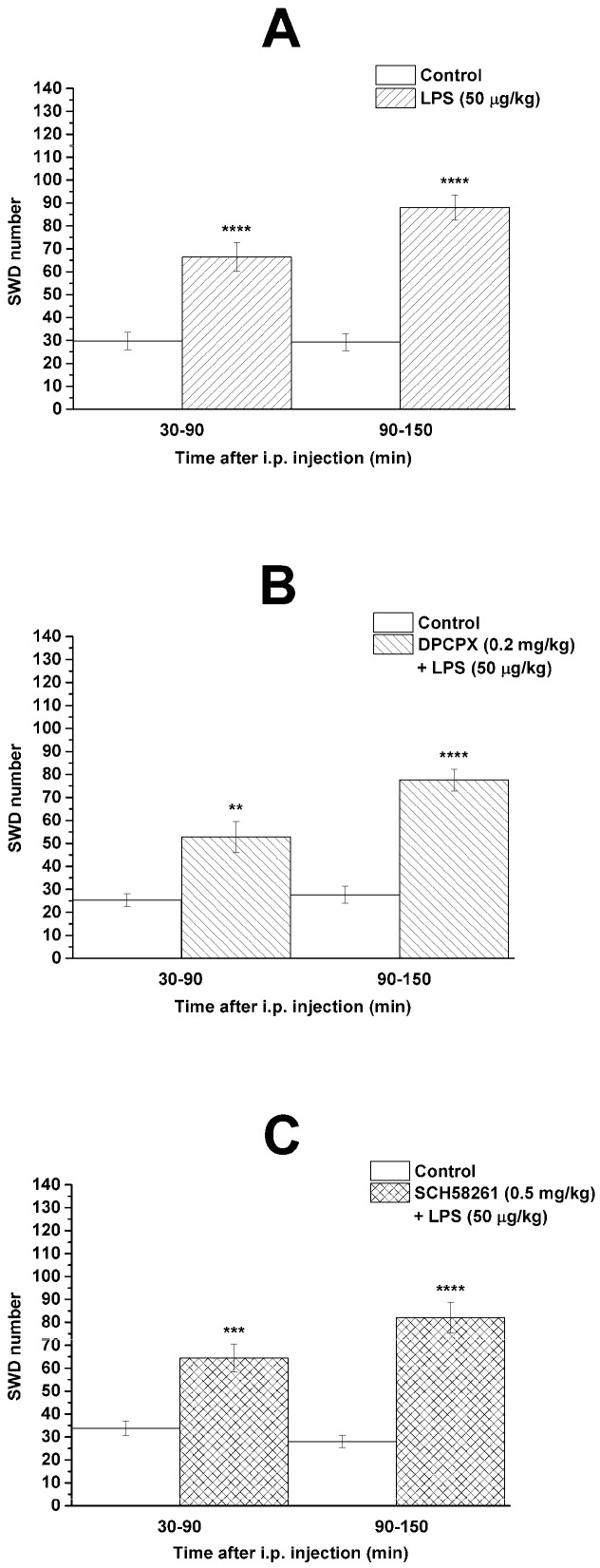
Effect of LPS (50 µg/kg) alone (**A**) and combined administration of DPCPX (0.2 mg/kg) with LPS (**B**) and SCH58261 (0.5 mg/kg) with LPS (**C**) on SWD number (group 1, group 2 and group 3, respectively). Abbreviations: DPCPX, 1,3-dipropyl-8-cyclopentylxanthine; i.p., intraperitoneal; LPS, lipopolysaccharide; SCH582361, 7-(2-phenylethyl)-5-amino-2-(2-furyl)-pyrazolo-[4,3-e]-1,2,4-triazolo[1,5-c]pyrimidine; SWD, spike-wave discharge. **: *p* < 0.01, ***: *p* < 0.001 and ****: *p* < 0.0001.

It has been previously demonstrated [23,34] that i.p. LPS (50 µg/kg) alone significantly enhanced the SWD number (group 1; Figure 2A; Table 1) between 30 and 150 min compared to the control. Combined administration of DPCPX (0.2 mg/kg) with LPS (50 µg/kg) (group 2) and SCH58261 (0.5 mg/kg) with LPS (50 µg/kg) (group 3) did not change the LPS-generated increase in SWD number (Figure 2B,C; Table 1).

### 3.2. Effect of DPCPX and SCH58261 on KEKS Treatment-Evoked Decrease in SWD Number after LPS Administration

**Figure 3:**
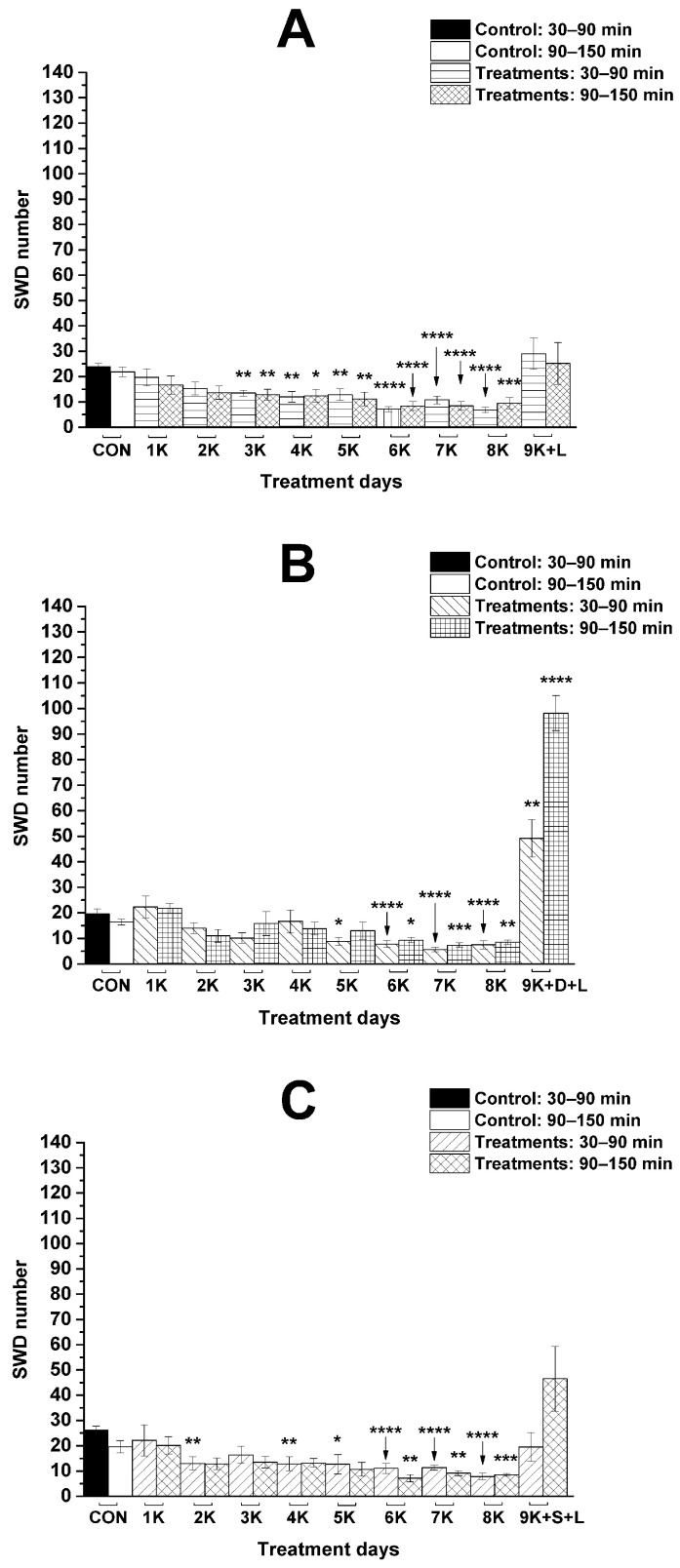
Influence of combined administration of KEKS-supplemented food (paste-like standard rodent chow containing 10% ketone ester/KE, 10% ketone salt/KS and 1% saccharin) with LPS (50 µg/kg) (**A**), with DPCPX (0.2 mg/kg) and LPS (**B**) and with SCH58261 (0.5 mg/kg) and LPS (**C**) on SWD number (groups 4, 5, and 6, respectively). Abbreviations: 1K, first day of KEKS food administration, 2K, second day of KEKS food administration and so on; 9K + D + L, 9th day of KEKS food administration and co-administration of i.p. DPCPX (0.2 mg/kg) and LPS (50 µg/kg); 9K + L, 9th day of KEKS food administration and co-administration of i.p. LPS (50 µg/kg); 9K + S + L, 9th day of KEKS food administration and co-administration of i.p. SCH58261 (0.5 mg/kg) and LPS (50 µg/kg); CON, control; SWD, spike-wave discharge. *: *p* < 0.05, **: *p* < 0.01, ***: *p* < 0.001 and ****: *p* < 0.0001.

As it was demonstrated previously, KEKS-supplemented food abolished the influence of i.p. LPS (50 µg/kg) alone on SWD number [23,34] (Figure 2A) between 30 and 150 min on the ninth KEKS day [19] (group 4; Figure 3A; Table 2), compared to control levels. Nevertheless, on the ninth day of KEKS food administration, when this treatment was combined with i.p. DPCPX (0.2 mg/kg) and LPS (50 µg/kg) (group 5; Figure 3B; Table 2), the SWD number significantly increased, compared to the control. Thus, administration of DPCPX abolished the alleviating effect of KEKS food on the LPS-evoked increase in SWD number (Figure 3A,B). Combined administration of SCH58261 (0.5 mg/kg) and LPS (50 µg/kg) with KEKS food (on the ninth day of KEKS food administration) was not able to change the beneficial effect of KEKS food on the LPS-generated increase in SWD number between 30 and 90 min (group 6; Figure 3C; Table 2). Nevertheless, SCH58261 only decreased, but did not abolish the KEKS food-generated beneficial effect on the LPS-evoked increase in SWD number between 90 and 150 min on the ninth KEKS food administration day (group 6; Figure 3C; Table 2).

After the combined i.p. administration of DPCPX (0.2 mg/kg) with LPS (50 µg/kg) and SCH58261 (0.5 mg/kg) with LPS (50 µg/kg) (groups 5 and 6, respectively) on the ninth day of KEKS food administration, the average SWD time did not change significantly (control/KEKS food + DPCPX + LPS: 7.5 ± 0.21 s/7.6 ± 0.33 s, *p* > 0.9999; control/KEKS food + SCH58261 + LPS: 7.6 ± 0.42 s/7.5 ± 0.29 s, *p* > 0.9999). As the average SWD duration did not change after these treatments and the SWD number significantly changed (increased) only after i.p. DPCPX + LPS (group 5; Figure 3B; Table 2), whereas the SWD number did not change significantly after i.p. SCH58261 + LPS (group 6; Figure 3C; Table 2) on the ninth KEKS food administration day, compared to the control, alterations in the total time of SWDs could be different in these two groups. Indeed, the total time of SWDs increased after the combined administration of DPCPX with LPS (control/KEKS food + DPCPX + LPS; 145.3 ± 14.1 s/372.1 ± 60.9 s, *p* < 0.0001), but the total time of SWDs did not change significantly after co-administration of SCH58261 with LPS (control/KEKS food + SCH58261 + LPS; 198.3 ± 15.9/143.8 ± 39.3 s, *p* = 0.83) on the ninth day of KEKS food treatment between 30 and 90 min, compared to the control.

### 3.3. KEKS-Generated Changes in Blood R-βHB and Glucose Levels and Body Weight

**Figure 4:**
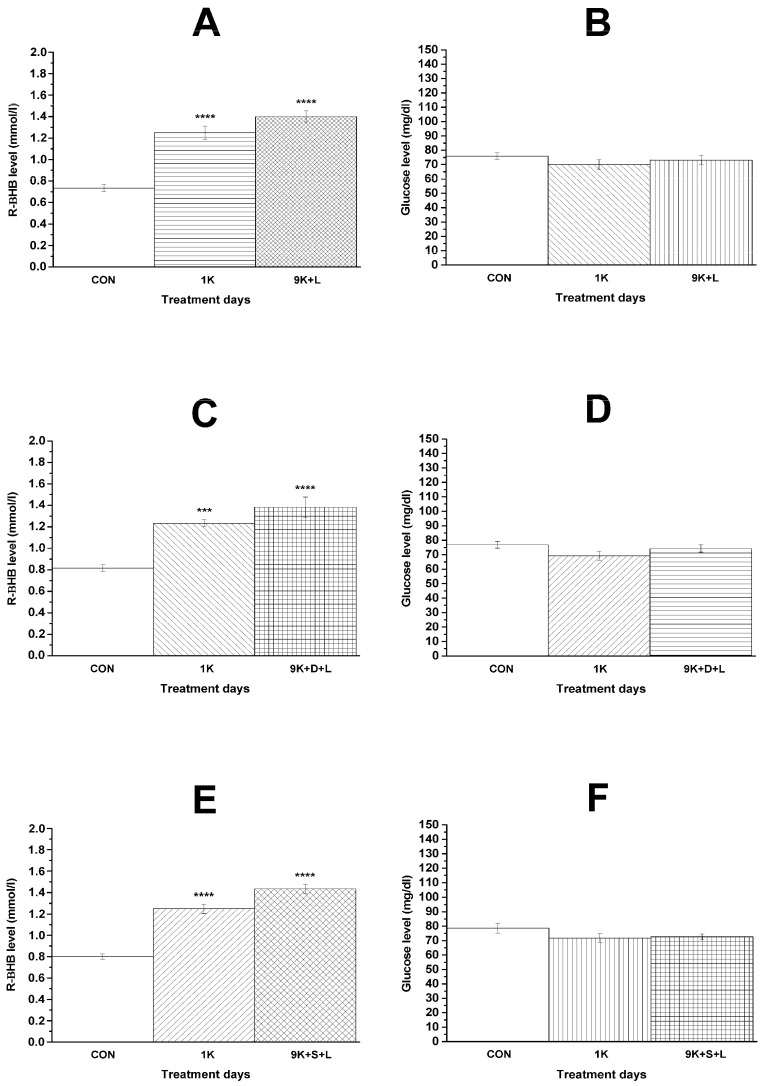
Changes in blood R-βHB and glucose levels on the first day of KEKS-supplemented food (10% ketone ester/KE, 10% ketone salt/KS and 1% saccharin in paste-like standard rodent chow) administration (1K; (**A**–**F)**) and on the ninth day of KEKS food administration combined with i.p. LPS (50 µg/kg) (9K + L; **A**,**B**), with i.p. DPCPX (0.2 mg/kg) and LPS (9K + D + L; **C**,**D**) and with i.p. SCH58261 (0.5 mg/kg) and LPS (9K + S + L; **E** and **F**), compared to the control (CON; groups 4, 5, and 6, respectively). ***: *p* < 0.001 and ****: *p* < 0.0001.

It was demonstrated that KEKS food treatment effectively increased the blood R-βHB level not only after the first KEKS food treatment, but also on the ninth day of KEKS food administration, independently of the combination of i.p. injections (LPS alone, DPCPX + LPS, SCH58261 + LPS; groups 4, 5, and 6; Figure 4A,C,E, respectively), compared to control levels. Nevertheless, the level of blood glucose was unchanged (groups 4, 5, and 6; Figure 4B,D,F, respectively) (Table 3).

The body weight of animals in the three KEKS treated groups (groups 4, 5, and 6) did not change significantly compared to the control (control/treated; group 4: 327.7 ± 3.63 g/323.8 ± 5.26 g, *p* = 0.5062; group 5: 334.7 ± 5.37 g/331.0 ± 6.68 g, *p* = 0.5344; group 6: 326.2 ± 6.56 g/323.5 ± 6.71 g, *p* = 0.8599). Similarly to our previous study [19], food intake was not investigated in the recent study. Nevertheless, the unchanged body weight of animals in groups 4, 5, and 6 suggests that KEKS food treatment did not exert its influence on the SWD number and SWD time through insufficient food intake or calorie restriction.

## 4. Discussion

In this study, we further validated our previous result that KEKS-supplemented food (KEKS food) is able to decrease the LPS-induced increase in SWD number [19] and provided new evidence that this alleviating effect of KEKS food may be mediated by the adenosinergic system, likely through A1Rs.

It has been demonstrated that IL-1β, LPS, and prostanoids (such as PGE_2_) can trigger cortical hyperexcitability [22,25]. Thus, LPS-generated inflammatory processes can intensify excitation in the hyperexcitable focal cortical zone (peri-oral region of the somatosensory cortex: cortical focus of absence epilepsy genesis) through proinflammatory cytokines in WAG/Rij rats, thereby increasing the SWD number [18,20,23,34] (Figure 2A). LPS has a role in the activation of NOD-like receptor pyrin domain 3 (NLRP3) inflammasome, TLR4 and release of IL-1β and TNF-α (tumor necrosis factor-α) [22,37,38], whereas KEKS food can inhibit the NLRP3 inflammasome and release of proinflammatory cytokines (such as IL-1β) through increased βHB level (ketosis) [16,39]. Moreover, KEKS food-evoked increase in βHB levels can also inhibit the activity of different parts of signaling pathways via the TLR4, IL-1 receptor, TNF-α, COX-2, and NF-κB (nuclear factor-κB) through, for example, hydroxyl-carboxylic acid receptor 2 (HCAR2 receptor) [40,41,42]; therefore, it may decrease the SWD number in WAG/Rij rats. Indeed, KEKS food-evoked ketosis decreased the LPS-induced increase in SWD number likely through the inhibition of inflammatory processes in WAG/Rij rats [11,19] (Figure 3A).

Ketosis may increase the concentration of adenosine [30], and thereby can modulate the activity of adenosine receptors. It was also suggested that EKSs-generated alleviating effects on absence epileptic activity may be mediated through increased βHB level-evoked increase in adenosine level and A1R activity [11,19]. Indeed, EKSs-induced increase in βHB level can increase the level of not only extracellular ATP, but also adenosine [4,43,44]. Thus, adenosine may evoke hyperpolarization of neuronal membranes and decrease neuronal activity through, for example, A1R-mediated opening of ATP-sensitive potassium channels and synaptic inhibition [4,29,44,45] resulting in both moderate hyperexcitability in the cortical focus and a decreased SWD number in WAG/Rij rats. It was also demonstrated that the activation of A2ARs increased the SWD number in WAG/Rij rats [28,46], suggesting that A2ARs are not able to modulate the alleviating influence of EKSs-generated ketosis on the number of spontaneously developed SWDs in WAG/Rij rats [11].

It has been previously demonstrated that administration of a non-selective adenosine receptor antagonist theophylline (i.p. 5 and 10 mg/kg) and i.p. 1 mg/kg SCH58261 decreased the SWD number [46,47] in WAG/Rij rats. In another rat model of human absence epilepsy GAERS (Genetic Absence Epilepsy Rats from Strasbourg), similar results were demonstrated, where another non-selective adenosine receptor antagonist caffeine (i.p. 1, 2.5, 5 and 10 mg/kg) decreased both the SWD number and total SWD time, and the A2AR antagonist DMPX (3,7-dimethyl-1-propargylxanthine; i.p. 0.15 and 0.3 mg/kg) generated a modest decrease in the total SWD time [48]. It was also demonstrated that i.p. 0.5 mg/kg DPCPX increased the SWD number in WAG/Rij rats [11], whereas a different A1R antagonist 8-CPT (8-cyclopentyl-1,3-dimethylxanthine; i.p. 0.625 and 3 mg/kg) decreased the SWD number in GAERS [48]. Based on the above results, we can conclude that A1Rs are able to generate a different modulatory influence on absence epilepsy genesis in these models. Consequently, theoretically, ketosis-generated effects on a number of spontaneously developed SWDs may be different in WAG/Rij rats and GAERS, at least through the adenosinergic system. Indeed, despite that, a ketogenic diet increased the blood βHB level in GAERS, it was not able to alter the SWD number [49]. However, new studies are needed to reveal the reason(s) of the different influences of A1R antagonism on absence epileptic activity in WAG/Rij rats and GAERS (e.g., investigation of A1R expression and distribution in brain areas implicated in absence epilepsy genesis in both rat strains).

It has been demonstrated that adenosine is a key anti-inflammatory mediator [50]. In the central nervous system, adenosine exerts its effects on inflammatory processes mainly through A1Rs and A2ARs [51]. A1Rs and A2ARs are expressed in astrocytes, oligodendrocytes, and microglia modulating inflammatory processes [50,52]. For example, both an A1R agonist (2-chloro-N^6^-cyclopentyl-2’-deoxyadenosine) and an A2AR antagonist (8-chloro-9-ethyl-2-phenethoxyadenine) alleviated the neuroinflammation (which inflammation was evoked by a pro-inflammatory cytokine cocktail, containing TNF-α, IL-1β, and interferon-gamma/IFN-γ). Moreover, this A2AR antagonist also showed anti-oxidant properties in mixed glial cells and after intracerebroventricular injection of 10 µg LPS [51]. These results suggest that activation of A1Rs and inhibition of A2ARs may evoke alleviating (anti-inflammatory) effects on neuroinflammatory processes. Indeed, activation of A1Rs decreased both the astrocyte proliferation and the excessive activation of microglial cells, thereby attenuating neuroinflammation, whereas an increase in microglial activity and neuroinflammation was demonstrated in A1R knockout mice [53,54,55]. Moreover, A1R activation can attenuate LPS-induced inflammation through inhibition of hypoxia-inducible factor 1 accumulation, thereby downregulation of genes involved in inflammatory processes (e.g., inducible nitric oxide synthase, iNOS) in astrocytes [56]. Activation of A1Rs may also mitigate the harmful influence of ROS (reactive oxygen species) on brain cells [26]. It has also been demonstrated that administration of SCH58261 reduced the level of IL-1β, IL-6, iNOS, and TNFα, whereas an A2AR agonist (CGS21680) increased the level of cytokines in microglial cells [57]. A2AR antagonists (e.g., SCH58261) decreased the LPS-evoked activation of microglia and secretion of IL-1β in microglial cells [58,59]. A2AR activation can increase the activation and proliferation of astrocytes [60,61]. Moreover, activation of A2ARs in microglial cells can increase not only the activity of nitric oxide synthase (NOS) and the level of COX2 expression (one of the enzymes of prostaglandin/PGE synthesis), but also the release of cytokines, PGE_2,_ and NO [62,63]. Consequently, activation of A1Rs may attenuate, whereas activation of A2ARs may enhance the neuroinflammatory processes and their pathological consequences [56,59,64]. All of the above results suggest that KEKS food-evoked decrease in an LPS-generated increase in SWD number are likely modulated by A1Rs, at least in WAG/Rij rats (Figure 3A,B). However, it was also suggested that A2ARs can also evoke peripheral anti-inflammatory effects [50,65]; adenosine may decrease LPS-induced cytokine production through A2ARs [27]; activation of A2ARs may inhibit the iNOS expression and NO production [66]; and inhibition of A2ARs did not abolish, but somewhat decreased the KEKS food-evoked alleviating effect on LPS-generated increase in SWD number between 90 and 150 min (Figure 3C). Thus, the beneficial effect of KEKS food-generated A2AR activation on LPS-induced neuroinflammation thereby on increased SWD number cannot be excluded entirely.

## 5. Conclusions

Our results strengthened the potential of ketogenic supplements, such as KEKS-supplemented food, for the treatment of epilepsy through the inhibition of inflammatory pathways. In relation to the mechanism of action, it is likely that KEKS-induced ketosis modulated A1Rs to alleviate the neuroinflammation-induced increase in SWD number. Thus, theoretically, co-administration of EKSs and different modulators of adenosinergic systems (e.g. adenosine receptors) may allow us to develop promising therapeutic tools in the treatment of not only epilepsy, but also inflammation-evoked neurodegenerative diseases. However, further studies are needed to reveal molecular signaling between EKSs-evoked alleviating effects on the SWD number, neuroinflammation, and the adenosinergic system in different cells and brain areas implicated in absence epilepsy genesis.

## Figures and Tables

**Table 1:** Effect of LPS alone, and combined administration of DPCPX with LPS and SCH58261 with LPS on SWD number.

Treatments	Figure 2A (Group 1)		Figure 2B (Group 2)		Figure 2C (Group 3)	
	SWD Number (Mean ± SEM; Level of Significance/*p*-Value)					
	30–90 min	90–150 min	30–90 min	90–150 min	30–90 min	90–150 min
Control (CON)	29.8 ± 3.85	29.3 ± 3.71	25.3 ± 2.84	27.7 ± 3.79	33.8 ± 3.23	28.0 ± 2.74
LPS (50 µg/kg)	66.4 ± 6.31 ****/<0.0001	88.0 ± 5.55 ****/<0.0001	-	-	-	-
DPCPX (0.2 mg/kg) + LPS (50 µg/kg)	-	-	52.8 ± 6.78 **/<0.0037	77.6 ± 4.71 ****/<0.0001	-	-
SCH58261 (0.5 mg/kg) + LPS (50 µg/kg)	-	-	-	-	64.5 ± 6.09 ***/0.0009	82.0 ± 6.66 ****/<0.0001

Abbreviations: CON, control; DPCPX, 1,3-dipropyl-8-cyclopentylxanthine; i.p., intraperitoneal; LPS, lipopolysaccharide; SCH582361, 7-(2-phenylethyl)-5-amino-2-(2-furyl)-pyrazolo-[4,3-e]-1,2,4-triazolo[1,5-c]pyrimidine; SWD, spike-wave discharge. **: *p* < 0.01, ***: *p* < 0.001 and ****: *p* < 0.0001.

**Table 2:** Influence of KEKS-supplemented food (paste-like standard rodent chow containing 10% ketone ester/KE, 10% ketone salt/KS and 1% saccharin) on SWD number between first (1K) and 8th (8K) day of administration and on 9th day of KEKS food (9K) administration combined with i.p. LPS (L) (9K + L), i.p. DPCPX (D) and LPS (9K + D + L), as well as i.p. SCH58261 (S) and LPS (9K + S + L), compared to control (CON).

Treatments	Figure 3A (Group 4)		Figure 3B (Group 5)		Figure 3C (Group 6)	
	SWD Number (Mean ± SEM; Level of Significance/*p*-Value)					
	30–90 min	90–150 min	30–90 min	90–150 min	30–90 min	90–150 min
Control (CON)	23.8 ± 1.36	21.9 ± 1.83	19.7 ± 1.98	16.4 ± 1.18	26.2 ± 1.61	19.6 ± 2.44
1st KEKS treatment (1K)	19.7 ± 3.38 ns/0.6999	16.7 ± 3.56 ns/0.5327	22.3 ± 4.19 ns/0.8824	21.8 ± 1.89 ns/0.4668	22.1 ± 6.18 ns/0.8818	20.2 ± 3.46 ns/0.9996
2nd KEKS treatment (2K)	15.3 ± 2.49 ns/0.0522	13.7 ± 2.70 ns/0.0623	14.0 ± 2.07 ns/0.2113	11.0 ± 2.46 ns/0.2397	13.0 ± 2.58 **/0.0030	12.8 ± 2.34 ns/0.1862
3rd KEKS treatment (3K)	13.5 ± 1.18 **/0.0015	12.8 ± 2.14 **/0.0051	10.2 ± 1.99 ns/0.1100	15.8 ± 4.71 ns/0.9986	16.3 ± 3.38 ns/0.0526	13.5 ± 2.25 ns/0.3380
4th KEKS treatment (4K)	12.0 ± 2.03 **/0.0021	12.3 ± 2.51 */0.0132	16.7 ± 4.55 ns/0.8773	13.8 ± 2.52 ns/0.9141	12.8 ± 2.79 **/0.0020	13.2 ± 1.78 ns/0.2007
5th KEKS treatment (5K)	12.8 ± 2.30 **/0.0073	11.2 ± 2.70 **/0.0089	8.8 ± 1.56 */0.0104	13.0 ± 3.34 ns/0.6795	12.7 ± 3.84 */0.0130	10.7 ± 2.75 ns/0.1382
6th KEKS treatment (6K)	7.2 ± 0.95 ****/<0.0001	8.3 ± 1.78 ****/<0.0001	7.8 ± 1.42 ****/<0.0001	9.3 ± 1.09 */0.0123	11.0 ± 2.01 ****/<0.0001	7.2 ± 1.45 **/0.0010
7th KEKS treatment (7K)	10.8 ± 1.52 ****/<0.0001	8.5 ± 1.73 ****/<0.0001	5.7 ± 0.92 ****/<0.0001	7.3 ± 0.92 ***/0.0005	11.3 ± 0.96 ****/<0.0001	9.2 ± 1.01 **/0.001
8th KEKS treatment (8K)	6.8 ± 1.01 ****/<0.0001	9.5 ± 2.20 ***/0.0002	7.5 ± 1.57 ****/<0.0001	8.5 ± 0.96 **/0.0054	7.8 ± 1.30 ****/<0.0001	8.5 ± 0.56 ***/0.0005
9th KEKS treatment (9K + L)	29.0 ± 6.16 ns/0.8988	25.2 ± 8.26 ns/0.9712	-	-	-	-
9th KEKS treatment (9K + D + L)	-	-	49.2 ± 7.31 **/0.0032	98.2 ± 6.86 ****/<0.0001	-	-
9th KEKS treatment (9K + S + L)	-	-	-	-	19.5 ± 5.71 ns/0.9117	46.5 ± 12.79 ns/0.0663

Abbreviations: ns, non-significant; SWD, spike-wave discharge. *: *p* < 0.05, **: *p* < 0.01, ***: *p* < 0.001 and ****: *p* < 0.0001.

**Table 3:** Influence of KEKS supplemented food (paste-like standard rodent chow containing 10% ketone ester/KE, 10% ketone salt/KS and 1% saccharin) on blood R-βHB and glucose levels on first (1K) day of administration and on 9th day of KEKS food (K) administration combined with i.p. LPS (L) (9K + L), i.p. DPCPX (D) and LPS (9K + D + L), as well as i.p. SCH58261 (S) and LPS (9K + S + L), compared to control (CON).

Treatments	Figure 4A,B (Group 4)		Figure 4C,D (Group 5)		Figure 4E,F (Group 6)	
	Blood Level of R-βHB and Glucose (Mean ± SEM; Level of Significance/*p*-Value)					
	R-βHB (mmol/L)	Glucose (mg/dL)	R-βHB (mmol/L)	glucose (mg/dL)	R-βHB (mmol/L)	Glucose (mg/dL)
Control (CON)	0.73 ± 0.03	75.83 ± 2.23	0.82 ± 0.03	76.83 ± 2.27	0.80 ± 0.03	78.67 ± 3.49
first KEKS treatment (1K)	1.25 ± 0.06 ****/<0.0001	70.17 ± 3.26 ns/0.3827	1.23 ± 0.03 ***/0.0006	69.17 ± 3.03 ns/0.1336	1.25 ± 0.04 ****/<0.0001	71.83 ± 2.94 ns/0.2445
9th KEKS treatment (9K + L)	1.40 ± 0.05 ****/<0.0001	73.17 ± 3.19 ns/0.7992	-	-	-	-
9th KEKS treatment (9K + D + L)	-	-	1.38 ± 0.09 ****/<0.0001	74.33 ± 2.57 ns/0.7842	-	-
9th KEKS treatment (9K + S + L)	-	-	-	-	1.43 ± 0.04 ****/<0.0001	72.67 ± 1.98 ns/0.3296

Abbreviations: R-βHB, R-beta-hydroxybutyrate. ***: *p* < 0.001 and ****: *p* < 0.0001.

## Data Availability

The data presented in this study are available on request from the corresponding author.

## Abbreviations

A1R	adenosine A_1_ receptor
A2AR	adenosine A_2A_ receptor
AcAc	acetoacetate
βHB	beta-hydroxybutyrate
COX	cyclooxygenase
DMSO	dimethyl sulfoxide
DPCPX	1,3-dipropyl-8-cyclopentylxanthine
EEG	electroencephalogram
IL-1β	interleukin-1β
iNOS	inducible nitric oxide synthase
i.p.	intraperitoneal
KE	ketone ester
KEKS	KE + KS
KS	ketone salt
LPS	lipopolysaccharide
NLRP3	NOD-like receptor pyrin domain 3
PGE_2_	prostaglandin E_2_
SCH58261	7-(2-phenylethyl)-5-amino-2-(2-furyl)-pyrazolo-[4,3-e]-1,2,4-triazolo[1,5-c]pyrimidine
SWD	spike-wave discharge
TLR4	Toll-like receptor 4
TNF-α	tumor necrosis factor-α
WAG/Rij	Wistar Albino Glaxo/Rijswijk
